# The Addition of Ferritin Enhanced the Prognostic Value of International Prognostic Index in Diffuse Large B‐Cell Lymphoma

**DOI:** 10.3389/fonc.2021.823079

**Published:** 2022-01-19

**Authors:** Ziyuan Shen, Shuo Zhang, Meng Zhang, Lingling Hu, Qian Sun, Chenlu He, Dongmei Yan, Jingjing Ye, Hao Zhang, Ling Wang, Weiying Gu, Yuqing Miao, Qinhua Liu, Changli Ouyang, Junfeng Zhu, Chunling Wang, Taigang Zhu, Shuiping Huang, Wei Sang

**Affiliations:** ^1^ Department of Epidemiology and Biostatistics, School of Public Health, Xuzhou Medical University, Xuzhou, China; ^2^ Department of Hematology, Affiliated Hospital of Xuzhou Medical University, Xuzhou, China; ^3^ Department of Hematology, Qilu Hospital of Shandong University, Jinan, China; ^4^ Department of Hematology, The Affiliated Hospital of Jining Medical University, Jining, China; ^5^ Department of Hematology, Taian Central Hospital, Taian, China; ^6^ Department of Hematology, The First People’s Hospital of Changzhou, Changzhou, China; ^7^ Department of Hematology, Yancheng First People’s Hospital, Yancheng, China; ^8^ Department of Hematology, The First Affiliated Hospital of Anhui Medical University, Hefei, China; ^9^ Department of Nuclear Medicine, Affiliated Hospital of Xuzhou Medical University, Xuzhou, China; ^10^ Department of Hematology, The First Affiliated Hospital of Bengbu Medical College, Bengbu, China; ^11^ Department of Hematology, Huai’an First People’s Hospital, Huaian, China; ^12^ Department of Hematology, The General Hospital of Wanbei Coal-Electric Group, Suzhou, China; ^13^ Center for Medical Statistics and Data Analysis, School of Public Health, Xuzhou Medical University, Xuzhou, China

**Keywords:** ferritin, DLBCL, International Prognostic Index, prognosis, nomogram

## Abstract

Diffuse large B-cell lymphoma (DLBCL) is a highly heterogeneous non-Hodgkin lymphoma, and the prognosis of DLBCL patients is widely affected by multivariables. Clinical-factors-based prognostic systems stratify the prognosis of DLBCL with certain limitations, and the value of ferritin on the prognosis of DLBCL is unclear. In this study, 225 cases were retrieved from 4 centers of Huaihai Lymphoma Working Group (HHLWG) as the derivation cohort, and 66 cases were from the other 6 centers of HHLWG as external validation cohort. X-Tile program divided ferritin into three groups when applying 175.00 and 391.90 μg/L as the optimal cutoff points. Based on multivariable analysis, ferritin appeared to be a stronger predictor. A total of three variables (ferritin, age, and lactate dehydrogenase) were included for the development of the nomogram. The C-indexes were 0.73 and 0.70 in the derivation and validation cohort, and the calibration curve showed the consistency between the nomogram prediction and the actual observation. In conclusion, Ferritin-based nomogram enhanced the prognostic value of IPI in DLBCL.

## Introduction

Diffuse large B-cell lymphoma (DLBCL) is the most common subtype of non-Hodgkin lymphoma (NHL) with high heterogeneity (1, 2). Cell origin, gene-based molecular stratification, immune markers, and other clinical variables pose great challenge to the DLBCL prognosis (3, 4). Clinical-characteristics-based International Prognostic Index (IPI) is widely used for DLBCL prognosis evaluation, but it failed to accurately classify variables such as age and lactate dehydrogenase (LDH). More importantly, with the addition of rituximab to anthracycline-containing therapy, its capacity to discriminate among risk groups was declined (5, 6). Several updated prognostic models have been developed and have greatly improved the prognostic accuracy of DLBCL (3, 6, 7). However, considering the high heterogeneity of DLBCL and advances of treatment, more variables such as nutritional markers, clinical factors, pathological characteristics, and genetic abnormalities are worthy of exploration to build more accurate prognostic system and to guide individualized treatment (8).

Nutritional markers are of great significance for the assessment of tumor prognosis. For example, obesity is associated with a 35%–40% increased risk of breast cancer recurrence and death (9). Ronald Wihal Oei et al. revealed that critical weight loss and low pretreatment hemoglobin were prognostic factors for survival in patients with nasopharyngeal carcinoma (10). Iron overload is usually associated with severe inflammation and pathological nutrition status. Some reports demonstrated that high serum ferritin level at diagnosis was a poor prognostic marker for some solid tumors including pancreatic cancer and head and neck cancer (11–13). In addition, Jun Zhou et al. found that high ferritin levels inversely correlate with survival for adult patients with hemophagocytic lymphohistiocytosis (HLH) (14). More specifically, a retrospective analysis showed that ferritin levels above 500 ng/ml could be an important marker for predicting poor survival outcomes in NHL (15). Da Jung Kim et al. revealed that high ferritin level (≥ 500 ng/ml) was an independent poor prognostic factor for progress-free survival (PFS) and overall survival (OS) in DLBCL patients with lower-risk IPI scores (16). However, few studies assessed the optimal cutoff point of ferritin level in the prognostic models for patients with DLBCL.

Different statistical methods have been used in predicting the outcomes of different diseases. Shaoxu Wu et al. made their prognosis for patients with bladder cancer by a nomogram (17, 18). The visual format of nomogram reflects a statistical prediction that can determine how many points are attributed for each variable value and the relative importance of predictors can be judged by the length of each line within the nomogram (19). X-Tile program can visualize the survival data and calculate the optimal cutoff point by dividing subgrouping. In addition, the associations can be determined by a variety of statistical tests, including the log-rank test for survival and means tests for associations between other marker data (20). In this study, continuous variables were processed by X-Tile program to determine the optimal threshold, and a nomogram was developed to stratify the prognosis for patients with DLBCL based on the levels of ferritin, other related nutritional markers, and baseline clinical factors.

## Materials and Methods

### Patients

From February 2015 to January 2021, a total of 291 patients with DLBCL and initial ferritin data were included in this study from HHLWG: 225 patients from 4 centers served as the derivation cohort, and 66 cases from the other 6 centers of HHLWG served as external validation cohort. All patients included in this study were with pathological diagnosis of DLBCL and treated with rituximab-based immunochemotherapy. Exclusion criteria were the following: (1) patients with other hematological malignancies; (2) special types of lymphoma (primary central nervous system lymphoma, primary mediastinal large B-cell lymphoma, and transformed DLBCL). The baseline characteristics included initial data of gender, age, extranodal involvement, performance status (21), presence of bulky disease (≥7.5cm), B symptoms, ferritin (Fer), lactate dehydrogenase (LDH), albumin (Alb), white blood cell count (WBC), hemoglobin (HGB), platelets (PLT), stage, cell of origin, and immunological markers. Follow-up was conducted through reviewing inpatient medical records and making phone calls. Overall survival (OS) was calculated as the interval between the time of diagnosis and death from any cause or the last follow-up. Study approval was obtained from the independent Ethics Committees of each center in HHLWG and met Helsinki Declaration.

### Statistical Analysis

Baseline clinical characteristics were described by variable type using median and interquartile range. Outliers were verified by the hospital medical record system. All cases had complete clinical information to avoid unnecessary bias. The value of ferritin was used as a continuous variable in the Mann–Whitney *U-*test to explore the difference between groups in different pathological groups. Continuous variables of ferritin, albumin, Body Mass Index (BMI), and age were divided into groups using the X-Tile program based on survival time (20). Kaplan–Meier analysis was used to explore the effect of pathological factors combined with ferritin level on survival, and log-rank test was performed for the difference between groups. Cox proportional hazard model was used to analyze the univariate association between clinical features and prognosis, with *p* < 0.05. The best prediction variable set was obtained by both stepwise regression, and Akaike Information Criteria (AIC) was used to evaluate the model (22). The Harrell’s concordance index and calibration curve of the model were calculated by bootstrap method according to the regression results, and validation was carried out in parallel (21). The number of self-sampling B was 500. The closer the concordance index (C-index) to 1, the better the prediction performance. Statistical analysis was conducted with SPSS software [version 19.0 (IBM, NY, USA)] and R software (version 4.2.0; http://www.Rproject.org).

## Results

### Clinical Characteristics

The characteristics of the patients are detailed in **Table 1**. The follow-up deadline was July 15, 2021, and the median follow-up time was 36.7 months in derivation cohort, and 23.1 months in validation cohort. At the end of follow-up, a total of 119 (40.90%) deaths occurred. The median age at diagnosis was 62 years, with 159 (54.60%) male and 168 (57.70%) patients were older than 60 years. Ann Arbor stage III/IV accounted for 66.60%.

**Table 1:** Characteristics of DLBCL patients in two cohorts.

Variables	Derivation cohort (n = 225)	Validation cohort (n = 66)
Age (year)	64 (55–71)	59 (47–67)
Gender: male	125 (55.50%)	34 (51.5%)
Stage: III/IV	144 (64.00%)	50 (75.7%)
IPI: LR/LIR	121 (53.70%)	30 (45.4%)
Bulky (≥7.5 cm)	27 (12.00%)	6 (9.10%)
B symptoms (presence)	48 (21.30%)	20 (30.30%)
ECOG (2–4)	44 (19.60%)	20 (30.30%)
CNS involvement	22 (9.80%)	9 (13.60%)
Fer (μg/L)	189.5 (100.60–381.50)	355.1 (132.8–719.0)
Alb (g/L)	38.1 (42.50–41.40)	35.95 (32.30–40.80)
WBC (×10^9^/L)	6.18 (4.70–7.95)	5.55 (4.30–6.72)
HGB (g/L)	121 (105–134)	110.5 (83.00–124)
PLT (×10^9^/L)	226.5 (162.00–281.00)	187 (117–242)
LDH (U/L)	232 (184–380)	318.5 (196–606)

Fer, ferritin; Alb, albumin; LDH, lactate dehydrogenase; HGB, hemoglobin; PLT, platelet; WBC, white blood cell count; IPI, International Prognostic Index; CNS involvement, central nervous system involvement.

### The Cutoff Points of Ferritin and Other Continuous Variables Calculated by X-Tile

**Figure 1:**
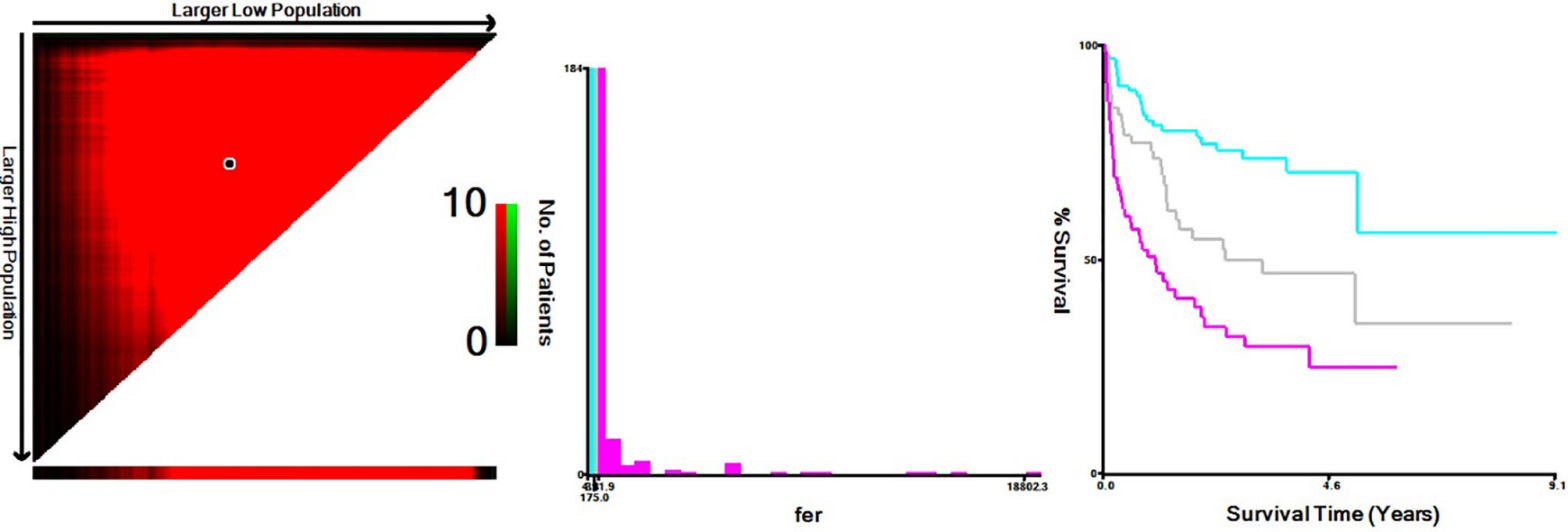
X-Tile data are displayed in a right-angled triangle grid, where each point represents a different cut point. The intensity of the color at each cutoff point indicates the strength of the association. X-Tile analysis of survival data from DLBCL patients reveals a continuous distribution based on ferritin. The plots show the c2 log-rank values produced when dividing the patients with two cut-points, producing high, middle, and low subsets.

Based on the X-Tile program, the maximum chi-squared points 22.13 and 25.39 were reached when applying 175.00 and 391.90 μg/L as the optimal cutoff points (*p* < 0.0001, **Figure 1**). Therefore, the DLBCL patients were divided into three subgroups for further analyses by using these cutoff points. Similarly, the optimal cutoff points for age, Alb, WBC, HGB, PLT, and LDH were 75 years, 40.30 g/L, 8 ×1 0^9^/L, 97 g/L, 205.50 × 10^9^/L, and 248 U/L.

### Prognostic Value of Ferritin With Clinicopathological Patterns in DLBCL

Mann–Whitney *U-*test was conducted for exploring continuous ferritin values on different subtypes, and the results suggested that ferritin levels between Ann Arbor I/II, III/IV and IPI LR/LIR, HIR/HR groups were significantly different (*p* < 0.01), but no significant difference was found in BCL-2, BCL-6, and CD5 groups.

**Figure 2:**
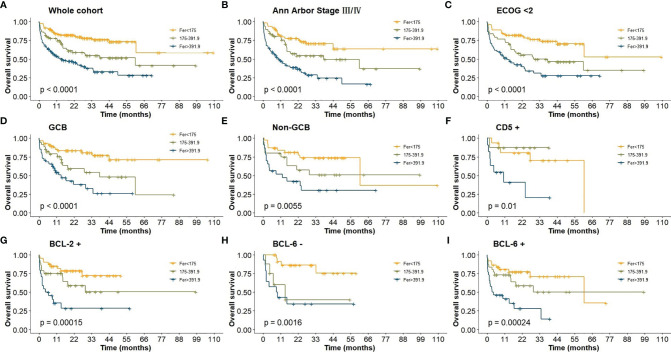
**(A)** OS of DLBCL patients in whole cohort; **(B)** OS of Ann Arbor Stage III/IV; **(C)** OS of ECOG (<2); **(D–E)** OS of cell-of-origin; **(F)** OS of CD5+; **(G)** OS of BCL-2+; **(H–I)** OS of BCL-6.

In the whole cohort, patients with high ferritin level (>391.90 μg/L) had poor survival with 5-year OS of only 28.2% (**Figure 2A**). Using cutoff values of 50% positive tumor cells for BCL-2 and BCL-6, 92 (31.60%) cases were positive for BCL-2, and 93 (31.90%) cases were positive for BCL-6. Thirty-six (12.4%) patients were with high Ki-67 score (≥ 0.9), and 81 (27.80%) were non-GCB. Kaplan–Meier analysis found that levels of ferritin could not re-stratify BCL-2, CD5, and Eastern Cooperative Oncology Group (ECOG) scores (≥2), and Ann Arbor Stage (I/II) (*p* > 0.05). However, the levels of ferritin could restratify the prognosis in BCL-2^+^, BCL-6, CD5^+^, cell of origin, ECOG score (<2), Ann Arbor Stage (III/VI), and IPI (*p* < 0.05, **Figure 2**).

### Univariable and Multivariable Analysis of DLBCL Patients

The effects of different clinical variables on OS were analyzed by univariable and multivariable analyses, and the results showed that the level of ferritin appeared to be a stronger predictor. Univariable analysis exhibited that Alb, HGB, PLT, and age were prognostic predictors (*p* < 0.05, **Table 2**). Following the model iterations in multivariable analysis, the final prognostic index consisted of three factors, as shown in **Table 2**. Ferritin was proved to be an adverse factor for the survival of DLBCL patients. Nevertheless, WBC in the current multivariable model was observed to be not predictive [*p* = 0.07, *HR* =1.52, 95% *CI* (0.97–2.37)].

**Table 2:** Prognostic factors of OS in the derivation cohort.

Univariable analysis				Multivariable analysis			
Variables	*HR*	95% *CI*	*p*	Variables	*HR*	95% *CI*	*p*
Fer				Fer			
<175	1			<175	1		
175–391.9	3.77	2.23–6.22	<0.01	175–391.9	2.05	1.17–3.59	0.01
>391.9	2.05	1.19–3.53	<0.01	>391.9	2.89	1.68–4.96	<0.01
LDH	3.56	2.24–5.65	<0.01	Age			
Alb	0.38	0.22–0.65	<0.01	<75			
HGB	0.48	0.32–0.73	<0.01	≥75	2.68	1.63–4.43	<0.01
Ann Arbor Stage	2.31	1.43–3.72	<0.01	LDH			
Age	2.12	1.32–3.42	<0.01	<248			
WBC	1.92	1.25–2.94	<0.01	≥248	2.14	1.28–3.57	<0.01
ECOG	2.54	1.26–5.14	0.01	WBC			
PLT	0.64	0.43–0.96	0.03	<8			
Ki-67	3.83	0.76–19.24	0.10	≥8	1.52	0.97–2.37	0.07
BCL-2	0.67	0.32–1.39	0.28	Ann Arbor Stage			
Gender	0.83	0.55–1.25	0.37	I/II			
COO	0.92	0.58–1.47	0.73	III/IV	1.50	0.89–2.53	0.13

Fer, ferritin; Alb, albumin; LDH, lactate dehydrogenase; HGB, hemoglobin; PLT, platelet; WBC, white blood cell count; COO, cell of origin.

### Development of Ferritin-Based Prognostic Nomogram and Validation

**Figure 3:**
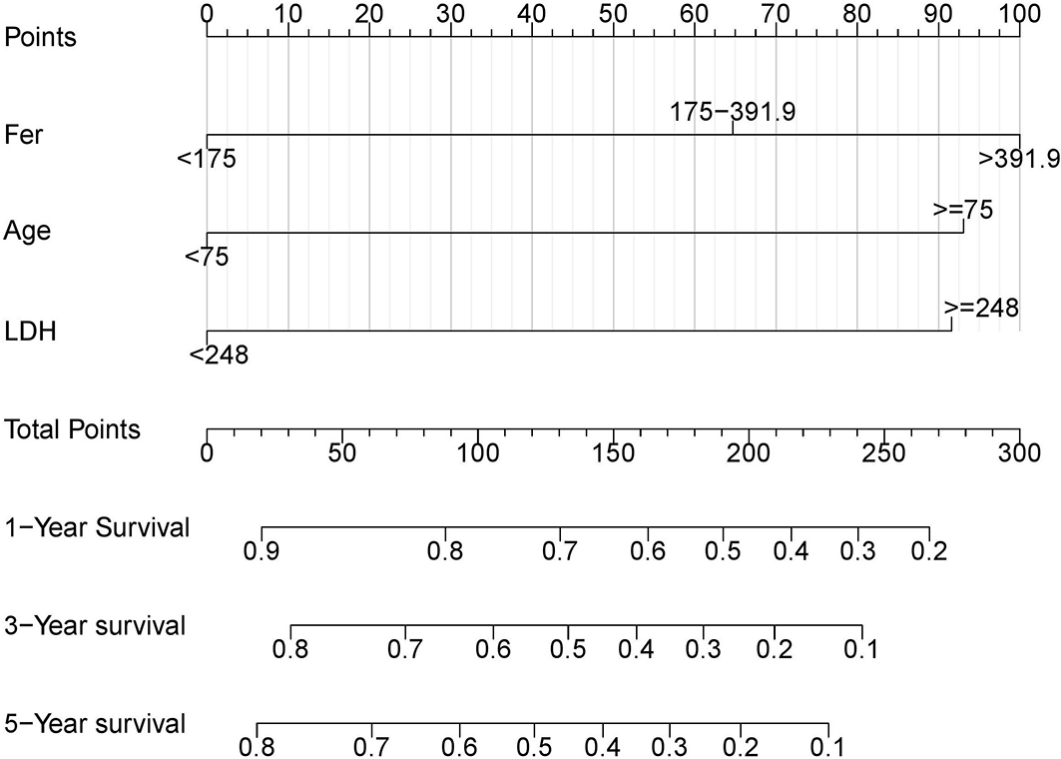
Nomogram for patients with DLBCL. The patient's clinical eigenvalues are placed on each variable axis and a line is drawn upward to determine the number of points gained for each variable value. The sum of these numbers lies on the total point axis, and then a line is drawn down on the survival axis to determine his overall survival probability.

Based on multivariable analysis, a prognostic nomogram was developed to predict 1-, 3-, and 5-year OS of DLBCL patients (**Figure 3**), and the Harrell’s concordance index and brier score (C-index = 0.73, Brier score = 0.17) were calculated between the predicted and the real outcome of the model for internal validation.

**Figure 4:**
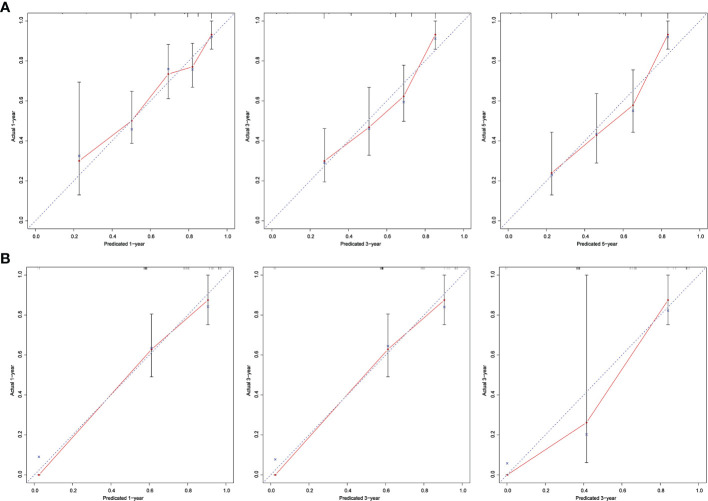
The red solid line represents the performance of the nomograph, and the higher the fitting degree with diagonal dotted line, the better the prediction effect. **(A)** Derivation cohort; **(B)** Validation cohort.

We further validated this nomogram externally by the calibration curve and computed the C-index and Brier score in an independent validation cohort of 66 patients (C-index = 0.70, Brier score = 0.22) in the external validation. The calibration curves were close to the ideal curves, suggesting that the predicted result and the actual outcome had a good consistency (**Figure 4**).

### Comparison of the Current Nomogram With IPI

**Figure 5:**
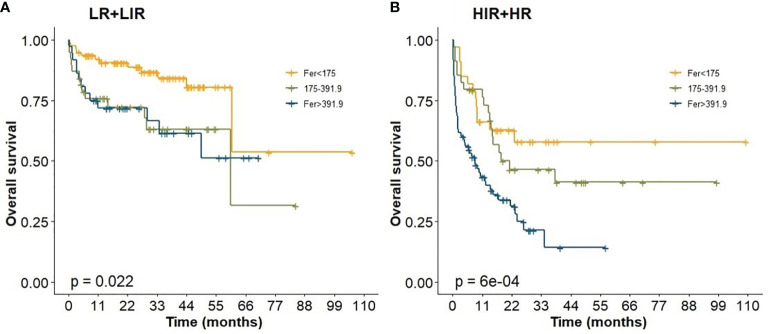
Prognostic impact of ferritin levels among patients in IPI. **(A)** Low risk/low intermediate risk group; **(B)** high intermediate risk/ high risk group.

In this study, all cases had complete data for all the variables required to calculate the IPI score. We analyzed the effect of ferritin levels on the prognosis in different IPI risk groups, and Kaplan–Meier analysis results are shown in **Figure 5**. Abnormal ferritin level was an adverse factor for patients in LIR/LR and HIR/HR groups in global comparisons (*p *<0.05, **Figure 5**). The 5-year OS for patients in different levels of ferritin in the LIR/LR group were 80.50%, 63.10%, and 51.20%, respectively (**Figure 5A**), and the 3-year OS in the HIR/HR group were 57.90%, 46.50%, and 21.50%, respectively (**Figure 5B**). Compared with IPI, the nomogram showed better accuracy in predicting survival of patients in both groups.

## Discussion

In this study, we assessed the level of ferritin to evaluate its prognostic value on DLBCL. Our research showed that patients with high level of ferritin had a poor prognosis. Based on model iteration, we established a prognosis nomogram for DLBCL patients, and the relative importance of the predictors could be determined by the length of the lines in the nomogram (23).

Nutritional markers affect the prognosis and survival of cancer patients. Indices such as the prognostic nutritional index (PNI) (24), albumin, BMI, and obesity have been well studied, and there is considerable evidence for their prognostic effects (25–29). Ferritin, an important indicator of metabolic level and nutrition, is associated with prognosis in end-stage liver disease, which can satisfactorily predict 11- and 90-day mortality (30).

A previous study in South Korea revealed that age, obesity, drinking habits, and glucose levels were significantly associated with women’s serum ferritin levels (31). Ferritin is detected at higher levels in the sera of many cancer patients, and the higher levels relate to aggressive disease and poor clinical outcome (32). In this study, we evaluated ferritin level using X-Tile program to seek more accurate cutoff point, and the results showed that the maximum chi-squared points of 22.13 and 25.39 were reached when applying 175.00 and 391.90 μg/L as the optimal cutoff points. Univariate Cox analysis showed that ferritin was a strong prognostic predictor of DLBCL. Elevated ferritin level (Fer ≥ 391.90 μg/L) was significantly correlated with prognosis. In addition, we used X-Tile program rather than the usual criteria to divide the age into two groups to achieve precise stratification. Multivariable analysis showed that when patients ≥75 years, the 3-year OS was only 28.10%.

R‐CHOP plus X regiments failed to improve OS in those patients with MYC and BCL‐2/BCL‐6 double expression/hit, activated B cells, and CD5-positive subtype. Previous studies have shown that different pathological immunophenotypes have an impact on outcomes (33, 34). In this study, ferritin level could restratify patients of CD5-positive group, and high levels of ferritin had a poor prognosis in both negative and positive BCL-6 groups (*p* < 0.05). High levels of ferritin in the GCB group also had a significant impact on the prognosis, but accurate stratification of ferritin in prognosis was not achieved in CD5- and BCL-2-negative groups.

The nomogram model demonstrated in this study included three variables: ferritin, age, and LDH. The specific DLBCL prognostic nomogram aimed to estimate the probability of 1-, 3-, and 5-year OS based on multivariable Cox proportional hazard models. Bootstrap resampling, C-index, and calibration curve were used to validate the model. The C-index was 0.73 between the predicted outcome and the real outcome of the model. The predicting clinical factors of the nomogram and original IPI were similar, with the former applying a refined categorization of age by X-Tile program. The C-index of the nomogram was higher than that of IPI, demonstrating a more obvious advantage than IPI. In conclusion, the addition of ferritin enhanced IPI for the prognosis of DLBCL. However, due to the inherent flaw of the retrospective design, further prospective studies need to be explored.

## Data Availability Statement

The raw data supporting the conclusions of this article will be made available by the authors, without undue reservation.

## Ethics Statement

The studies involving human participants were reviewed and approved by the independent Ethics Committees of each center in HHLWG and met Helsinki Declaration. The patients/participants provided their written informed consent to participate in this study.

## Author Contributions

WS and SH: designed this study. ZS and CH: analysis and interpretation. SZ, MZ, LH, QS, DY, JY, HZ, WG, YM, QL, CO, JZ, CW, and TZ: acquisition of data. CO provided the advices of this study. All authors contributed to the article and approved the submitted version.

## Funding

This study was funded by the Natural Science Foundation of Jiangsu Province, Grant/Award Number BK20171181; Jiangsu Key Research and Development Project of Social Development, Grant/Award Number BE2019638; and Young Medical Talents of Jiangsu Science and Education Health Project, Grant/Award Number QNRC2016791.

## Conflict of Interest

The authors declare that the research was conducted in the absence of any commercial or financial relationships that could be construed as a potential conflict of interest.

## Publisher’s Note

All claims expressed in this article are solely those of the authors and do not necessarily represent those of their affiliated organizations, or those of the publisher, the editors and the reviewers. Any product that may be evaluated in this article, or claim that may be made by its manufacturer, is not guaranteed or endorsed by the publisher.
